# A novel approach to locomotion learning: Actor-Critic architecture using central pattern generators and dynamic motor primitives

**DOI:** 10.3389/fnbot.2014.00023

**Published:** 2014-10-02

**Authors:** Cai Li, Robert Lowe, Tom Ziemke

**Affiliations:** ^1^Interaction Lab, School of Informatics, University of SkövdeSkövde, Sweden; ^2^Department of Computer and Information Science, Linköping UniversityLinköping, Sweden

**Keywords:** actor-critic, central pattern generators (CPG), reinforcement learning, locomotion control, NAO robot

## Abstract

In this article, we propose an architecture of a bio-inspired controller that addresses the problem of learning different locomotion gaits for different robot morphologies. The modeling objective is split into two: baseline motion modeling and dynamics adaptation. Baseline motion modeling aims to achieve fundamental functions of a certain type of locomotion and dynamics adaptation provides a “reshaping” function for adapting the baseline motion to desired motion. Based on this assumption, a three-layer architecture is developed using central pattern generators (CPGs, a bio-inspired locomotor center for the baseline motion) and dynamic motor primitives (DMPs, a model with universal “reshaping” functions). In this article, we use this architecture with the actor-critic algorithms for finding a good “reshaping” function. In order to demonstrate the learning power of the actor-critic based architecture, we tested it on two experiments: (1) learning to crawl on a humanoid and, (2) learning to gallop on a puppy robot. Two types of actor-critic algorithms (policy search and policy gradient) are compared in order to evaluate the advantages and disadvantages of different actor-critic based learning algorithms for different morphologies. Finally, based on the analysis of the experimental results, a generic view/architecture for locomotion learning is discussed in the conclusion.

## 1. Introduction

Locomotion modeling for robotics aims to endow a robot with the ability to propel itself in an environment. Traditional engineering approaches can model locomotion on a rigid-body robot with detailed modeling of a particular environment and body, such as zero moment point model and inverse kinematics model (for a review see Siciliano and Khatib, 2008). So traditional engineering approaches based locomotion models can work quite well in a constrained context but might have difficulties in adapting to different environments. However, a lot of modern robots are built on the basis of different animals, especially with distinct morphologies, e.g., fish robot (Marchese et al., 2013), worm robot (Ueno et al., 2014) and roboy humanoid (Pfeifer et al., 2014). None of their morphologies can be easily modeled. Therefore, bio-inspired approaches have been widely applied to model locomotion capabilities for such kind of robots (for a review see Ijspeert, 2008, Li, 2014), providing more flexibility focusing on the interaction with the environment and the emergence of different gaits. The dynamic systems theory was proposed by Thelen (1996) for emphasizing the importance of environmental interaction on the development of locomotor systems. The salient role of morphology (body) in the process of gait emergence was highlighted by Pfeifer and Bongard (2006). In order to model a locomotor system on a flexible body, we need to design (a) an interaction process involving the body and the environment; (b) a neural controller which can be adapted into a particular body following a design methodology and find a proper gait with this body in a particular environment. Therefore, in this article, key components (a body, an environment and a neural controller) of a locomotor system are highlighted and then a method for designing a robotic locomotion system adaptable to these components (based on learning) is proposed.

**Figure 1:**
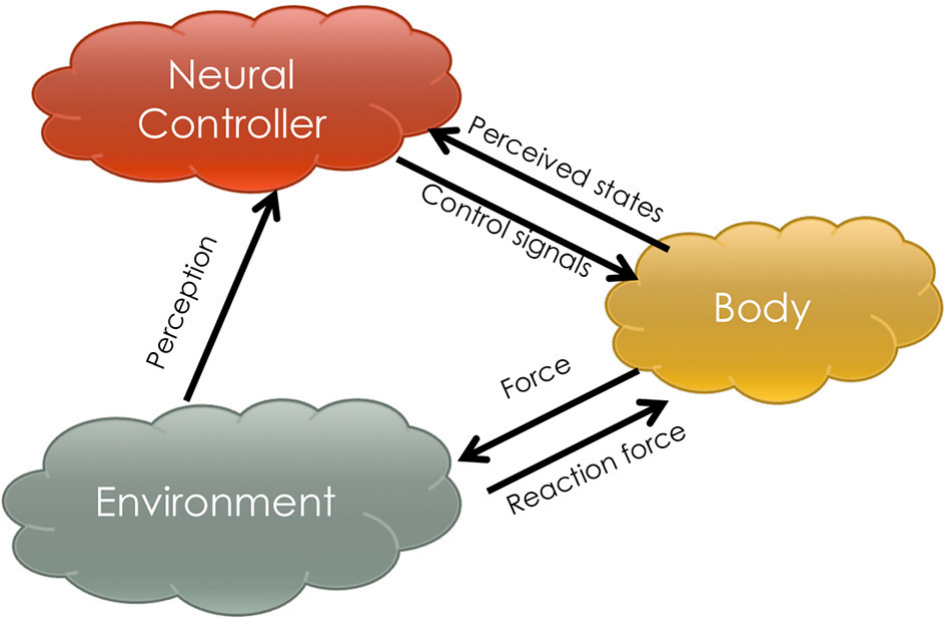
**This figure sketches the mechanism of three-component locomotion**. The arrows indicate the functional information flow amongst the components, the neural controller, the body and the environment.

Figure 1 shows a schema in which three key components of a locomotor system interact. The neural controller is a structure that assimilates perceptual information from the environment and the state space of the body. It can be highly complex with brain-like functions such as memorization, perception (e.g., vision), learnability and so forth. The body is a physical medium through which neural systems contact the environment. In most robotics scenarios, it refers to a high DOF mechanical structure. The environment is not specifically modeled but considered to have a significant role affecting locomotion. In the mechanism described by Figure 1, the neural controller emits control signals to the physical body and receives the perceived changes in body states. The body receives the control signals and acts on the environment. Between the body and the environment, the contact force (e.g., supporting force, tangential force.) determines the quality of locomotion. Then the neural controller can also perceive information from the environment to evaluate its own behavior in order to send out better control signals. However, without the complexity of modeling an environment and a body like traditional engineering approaches, a methodology is developed in this article for designing a neural controller that (a) has the ability to learn in the above-mentioned three component interaction process and (b) can be utilized on different morphologies. We provide an example instantiation and demonstrate its generalizability by evaluating it on two robot morphologies.

In terms of neural controllers, choosing central pattern generators (CPGs) is one mainstream bio-inspired solution to modeling quadrupedal locomotion (Degallier et al., 2008; Harischandra et al., 2011; Zhao et al., 2012). CPGs are neural circuits which are located in the spinal cord of vertebrates and able to generate rhythmic movement without sensory feedback (Orlovsky et al., 1999; Latash, 2008). According to Grillner et al. (2005) and Rybak et al. (2006), CPGs receive input from a lot of brain parts (e.g., basal ganglia and brainstem) and muscles. This means CPGs not only provide strong adaptation capabilities to a certain type of locomotion but also are useful to explore fundamental locomotion principles for transferring animal locomotion capabilities to robots. In most work, CPGs are used as sensory-input-dependent neural networks of which the output is considered as a force or trajectory generator. According to Ijspeert et al. (2013), there are two modeling objectives for locomotion capabilities: One is a baseline behavior which contains core foundational patterns for a type of motor ability, for example the coordination of joints. After this is accomplished, the second concerns dynamic adaptation: how the baseline patterns can adapt to complex and dynamical changes pertaining to the environment or the physical body. On the basis of these two objectives, Section 2.1 will introduce the design of *baseline behavior* and *dynamic adaptation*.

The concept of *motor primitives* has been defined by researchers from biology as sets of force-fields generated by muscle synergies (Mussa-Ivaldi, 1999). It is also coined as “building blocks of movement generation” by Schaal et al. (2004) from the perspective of motor control. A very important function of CPGs for adaptation is the “reshaping” function which reshapes the output of neural circuits into the required one (Rybak et al., 2006; Ijspeert et al., 2013). Dynamic motor primitives (DMPs) can be used as a universal morphed oscillator which can turn rhythmic output into desired ones by constraining search space to a period of rhythmic input (for details, please refer to Section 2). Therefore, in our work, DMPs are used to model the function of dynamics adaptation, a representation of “reshaping” function. The model of DMPs is broadly used for motor learning (Peters, 2007; Kober et al., 2012) in a supervised learning algorithms since it has a good capability of reshaping the output to different dynamics with linear regression techniques. Therefore, in this article, DMPs are chosen as an interface because of its learnability with RL algorithms.

As for the mechanism of interaction, reinforcement learning (RL) is a particularly effective mechanism for searching proper “reshaping” functions in locomotion learning, especially for robotic applications (Nakamura et al., 2007; Endo et al., 2008; Li et al., 2013b). Nakamura et al. (2007) and Li et al. (2013b) demonstrated CPG architectures without a general “reshaping” function. The former developed a “reshaping” function based on a predefined sum of several variables and the latter proposed a “reshaping” function based on the limited sum of sensory input. Proper joint dynamics cannot be properly found with limited “reshaping” function. In our work, periodic DMPs are used to avoid this problem. Endo et al. (2008) aimed to learn leg trajectories of a biped based on a detailed model of the body (inverse kinematics). However, in our work, the body does not need to be modeled, which makes our model able to be used on different morphologies. The novelty of our method on the implementation level focus on the emergence of a certain type of gait in an interactive learning process provided by RL. From the perspective of neuroscience, RL also sketches a bio-inspired function for integrating different perceptual information, especially the actor-critic mechanism regarding basal ganglia (Wiering and van Otterlo, 2012) emphasized by Grillner et al. (2008) in their biological CPG structure. On this basis, we consider using CPGs in an actor-critic RL schema (CPG-Actor-Critic) a suitable approach which we adopt after accomplishing the design of the CPG architecture. Section 2.2 will introduce the use of CPG-Actor-Critic.

Based on the above-mentioned perspective, in order to test the neural controller, two experiments were conducted on two different-morphology robots, the NAO robot (rigid body) and ghost dog (soft body), for learning crawling and running, respectively (Section 3). After that, the process of locomotion capabilities emerging from baseline behaviors which serve as “prior knowledge” is analyzed and the detailed analysis of dynamics adaptation is shown in terms of joint dynamics. Finally in Section 4, the conclusion regarding a generic neural structure of locomotion learning is drawn for the purpose of implementing locomotion learning in a robot.

## 2. Methods and theories

### 2.1. Design of the CPG architecture

CPGs have been investigated to model locomotion in many robotic applications and there are also many existing CPG models inspired by the biological underpinnings of various levels and species (Ijspeert, 2008). In this respect, oscillator models are the ones commonly used and with a lot of advantages. Firstly, oscillator models can be easily modeled by ordinary differential equations (ODEs). Secondly, dynamics of oscillators come from the topology of couplings in the oscillator network, which is based on the well-established dynamic systems theory, especially for the symmetric topology (Golubitsky and Stewart, 2003). So oscillator models have a strong mathematical background. Moreover, the focus of oscillator models is on how phase difference and synchronization of different oscillators can be determined by the topology couplings or frequencies of the oscillator populations, rather than rhythm generation. So an oscillator in the model is not a model of a neuron but rather works like a complete oscillation center. If each DOF of a robotic body is controlled by at least one oscillator, in terms of Grillner et al's (Grillner, 1985) assumptions, oscillator models can handle the problem of how each DOF is coordinated with others within a high-DOF body. According to Grillner et al's research (Grillner et al., 2008), CPGs biologically are able to assimilate two functions: dynamics adaptation and posture control. If each DOF of the robotic joint controlled by an oscillator is considered as an adaptive limit cycle, dynamics adaptation is the function of reshaping the limit cycle and the posture control points to the ability of shifting the oscillation center. There are also many oscillators with such functionalities (Righetti and Ijspeert, 2006; Pouya et al., 2010).

**Figure 2:**
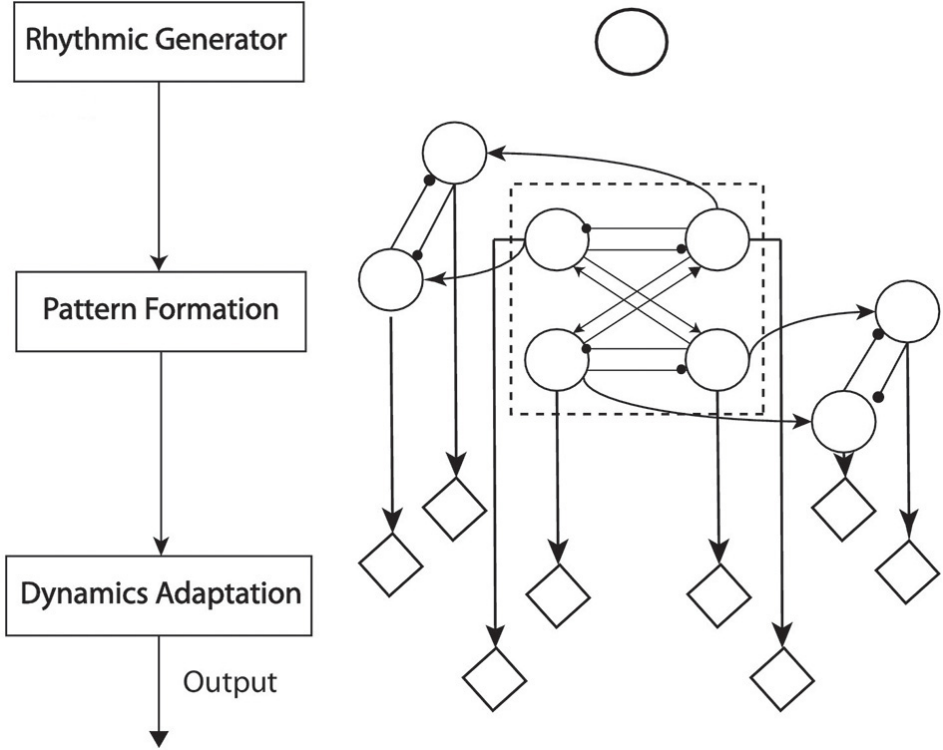
**Left:** The functional structure of CPG anatomy; each block represents one-layer functionality. **Right**: The neural structure of CPG employed for crawling. The single circle above most represents the RG layer as an oscillator. The recurrent neural network composed of connected circles represents the function of the PF layer. The diamonds represent functions of the DA layer. Within the PF-layer network, the four-cell network in the dash-line frame controls the rhythms of pitch motion for Shoulders and Hips. The other four outside the dash-line frame control the roll motion of Shoulders and Hips. The arrow-head lines represent in-phase oscillation (the phase difference between two oscillators is 0 or 2π). The dot-head lines represent anti-phase oscillation (the phase difference between two oscillators is π).

Rybak et al. (2006) uncovered a possible biological anatomy of CPGs (Figure 2 left). In this structure, the rhythm generator (RG) layer provides a primitive source of oscillatory signals. The pattern formation (PF) layer is a level on which all the RGs are mutually connected to form the phase-separated output. The dynamics adaptation (DA) represents the functions of motoneurons of which the output is sent directly to muscle fibers. In this layer, the output of PF layer is adapted into distinct dynamics in order to adapt to different environments or interactions. After reshaping the output of PF layer in DA layer, the RG itself turns out to be a “clocking” driver for CPGs. This three-layer architecture has been implemented to model walking behaviors (Li et al., 2013b; Nassour et al., 2013). This also matches the two objectives (Section 1) of locomotion modeling: from *baseline behaviors* to *dynamics adaptation*. RG and PF layers represent the architecture of baseline behavior. These two layers encode fundamental characteristics of one type of locomotion. For example, crawling is experimentally observed to be one type of locomotion featured by anti-phase movement of the ipisilateral limbs and in-phase movement of diagonal limbs, as coupling information (Righetti et al., 2008). The final layer is a layer of adapting baseline dynamics into desired locomotion.

#### 2.1.1. The method of designing the baseline behavior

**Figure 3:**
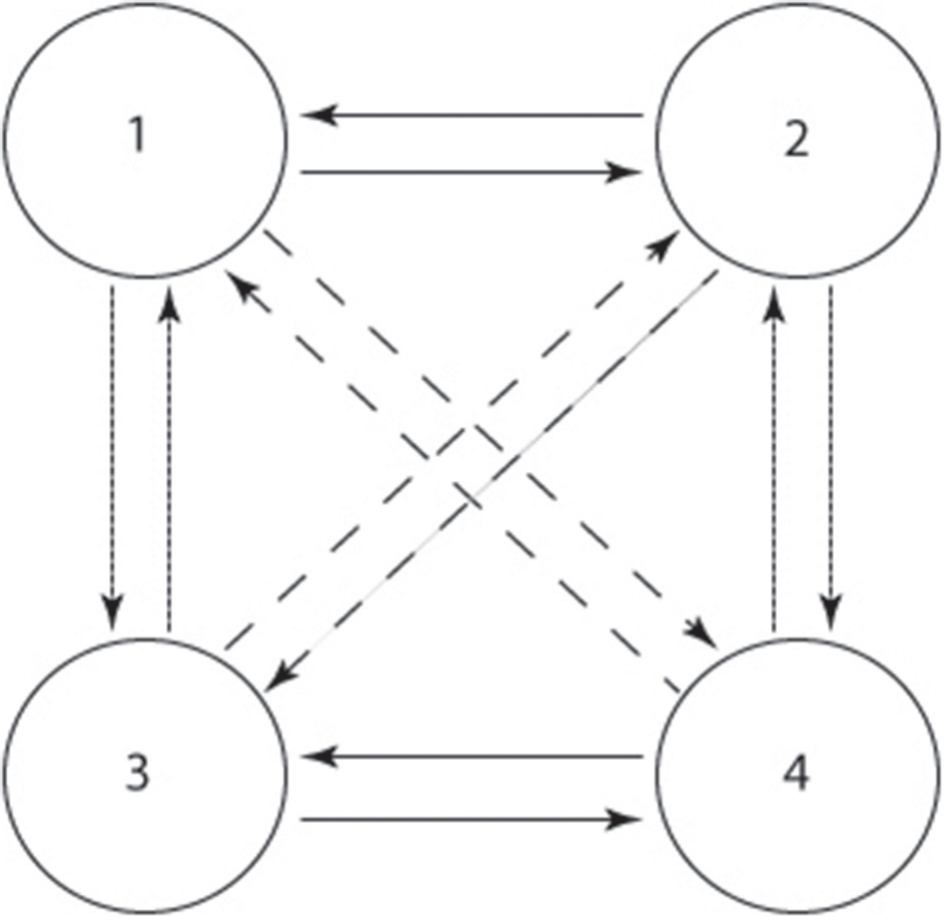
**The architecture of the four-cell CPG network**. The condense dashed, dashed, and straight arrow indicate ipsilateral, diagonal and contralateral couplings.

Golubitsky and Stewart (2003) propose an approach to designing the symmetric CPG topology based on the dynamic systems theory. A four-cell architecture (Figure 3) is widely used to coordinate the main joints (the joints attached to the main body, usually hip and shoulder joints) of a locomotor system (Degallier et al., 2008; Li et al., 2011). It is mathematically proved that this architecture can simulate the synchrony of different quadrupedal gaits, such as trot, walk, pace and gallop. If the couplings are changed, the transition amongst those gaits can be simulated too. On the other hand, as a minimal topology of CPGs, it can be extended to an eight-cell architecture by using zig-zag or cross coupling to generate all kinds of gaits for quadrupeds according to Golubitsky and Stewart (2003). Since all the quadrupedal animals have similar gaits and the four-cell architecture accounts for most such gaits (Righetti, 2008), it can be used to model the basic dynamics of each gait, including preliminary coupling, trajectory and frequency.

Before using this architecture, the existence of stable periodic solutions has to be determined according to the *H*/*K* theorem (Golubitsky and Stewart, 2003; Righetti, 2008) (for details, please refer to Supplementary Material). As examples of the *H*/*K* theorem, regarding the two gaits we are concerned with in this article, we can mathematically prove that the stable periodic solutions exist. Assume the nodes in Figure 3 control different joints respectively (1: left shoulder, 2: right shoulder, 3: left hip, 4: right hip. Knee and ankle joints are not controlled since it is not convenient for them to oscillate for a humanoid like NAO with big feet. All the other joints are synchronized with the correspondent joints controlled by the four-cell architectures, including roll and elbow joints), we can start to permute the symmetric characteristics of the architecture. In terms of crawling, a characteristic of crawling gaits is the anti-phase and in-phase relation of the ipsilateral limbs and diagonal limbs, respectively (for the detailed mathematical meanings of *H* and *K* group, please refer to Supplementary Material). So the spatial-temporal group *H_c_* and spatial group *K_c_* for crawling are:

$$\begin{array}{l} H_{c}:\left\{\left((12),(34),\frac{T}{2}\right),\left((13),(24),\frac{T}{2}\right),\left((14),(23),0\right)\right\}\\ K_{c}:\left\{\left((14),(23),0\right)\right\} \end{array}$$

where *T* is the period of one signal and $\frac{T}{2}$ is the phase shift in each group. Obviously *H_c_*/*K_c_* ≅ *Z*_2_ which is cyclic (Righetti, 2008) (also see Supplementary Material) and *K_c_* is an isotropy group. In terms of double-suspension gallop (front and rear feet are in phase respectively), the spatial-temporal group *H_g_* and spatial group *K_g_* of this gait are:

$$\begin{array}{l} H_{g}:\left\{\left((12),(34),0\right),\left((13),(24),a\right),\left((14),(23),a\right)\right\}\\ K_{g}:\left\{\left((12),(34),0\right)\right\} \end{array}$$

where *a* is the phase shift and *a* ∈ [0, *T*]. In the work described in this article, *a* is equal to 2. *H_g_*/*K_g_* ≅ *Z_m_* (*m* ≫ 2) which is cyclic too (For the proof please refer to Supplementary Material) and *K_g_* is an isotropy group.

Within the four-cell CPG network, each node can be modeled by an oscillator (e.g., numerical oscillators Li et al., 2013b or phase oscillators Pouya et al., 2010). The advantage of using phase oscillators is that the phase shift can be explicitly specified. Therefore, in this article, a standard phase oscillator is chosen:

$$\begin{array}{l} \;\dot{r_{i}}=a_{i}(R_{i}-r_{i})\\ \;\dot{W_{i}}=2\pi \omega_{i}+K_{i}\\ \;K_{i}=\sum_{j}w_{ji}\cdot r_{j}sin\left(W_{j}-W_{i}-P_{ji}\right)\\ \;\omega_{i}=\frac{\omega_{1i}}{e^{-100\cdot Aex_{i}}+1}+\frac{\omega_{2i}}{e^{100\cdot Aex_{i}}+1}\\ Aex_{i}=r_{i}\cdot sin\left(W_{i}+\frac{\pi }{2}\right)\\ \;A_{i}=r_{i}\cdot sin(W_{i}) \end{array}$$

where *A_i_* is the output of this phase oscillator and *Aex_i_* is the frequency control output. *r_i_* and *W_i_* are the amplitude and phase variables respectively. ω_*i*_ is the frequency of the oscillator with ω_1*i*_ and ω_2*i*_ controlling the ascending and descending frequency. *K_i_* is the connection term from the other oscillators to oscillator *i*. *w*_*ji*_ is the connection weight of from oscillator *j* to *i*. *W_j_* is the phase of oscillator *j* and *P*_*ji*_ is the phase difference from oscillator *j* to *i* (For example, in the four-cell network formed based on the *H*/*K* theorem, the groups ((13), (24), θ) (θ is the phase shift) are represented by setting *P*_31_ = θ (cell 1), *P*_42_ = θ (cell 2), *P*_13_ = −θ (cell 3) and *P*_24_ = −θ (cell 4), θ can be $\frac{T}{2}$ = π for crawling or *a* = 2 for galloping). *a_i_* and *R_i_* are the convergence rate and converged value of amplitude respectively. In our work, the parameter settings are as follows: *a_i_* = 50, *R_i_* = 1.0, *w*_1*i*_ = *w*_2*i*_ = 1.0.

In summary, corresponding to the RG and PF layer, a four-cell CPG network is utilized as a baseline motion generator to drive the motion of each joint (details are in Figure 2). This baseline motion generator has the capabilities to maintain structural stability according to group theory (Golubitsky and Stewart, 2003) and has been verified to generate basic patterns of both crawling and bipedal walking by adapting parameters *w*_1*i*_ and *w*_2*i*_ (Degallier et al., 2008; Li et al., 2011, 2012). Moreover, the prior knowledge about a specific gait is encoded in this baseline behavior generator to reduce the workload of gait learning. For example, taking advantage of the symmetric topology can reduce the number of DOFs which are to be learned/optimized so that the dimensions of further learning are lowered.

#### 2.1.2. Design of the dynamics adaptation function

With a baseline behavior, in this section we will discuss how to adapt it into a mature gait. This needs an architecture which can reshape/shift the baseline dynamics to achieve DA. There are two situations for DA in practical implementation: the body without (proper) sensors and with *useful* sensors. In a lot of cases, because of the mechanical design, some robots do not have useful sensors for certain types of locomotion gait. For example, the NAO robot does not have pressure sensors for crawling. So a general approach is required for the situation whether there are proper sensors or not. In fact, DA is a trivial function which involves how different perceptual/sensory information (e.g., sensory feedback) contributes to reshaping the dynamics of each DOF. Since there is no systematic approach to finding a reshaping mechanism regarding to different sensory feedback and robots also have different sensor configurations, in this article, DA only focuses on an abstract basic mathematical framework to achieve the same function of reshaping the dynamics based on the baseline behavior in order to alter the trajectories/dynamics of each joint. Therefore, an architecture which can modify the joint movement is required. According to Ijspeert et al. (2013)^1^, DMPs have two types of format: discrete and periodic. They differ in the forcing term *f*. In our work, since the task is to learn a rhythmic movement, the periodic DMPs are selected as a dynamics modifier and the mathematical model of periodic DMPs is:

(1) $$\begin{array}{l} \;\tau \dot{z_{i}}=\alpha \left(\beta (g_{i}-y_{i})-z_{i}\right)+amp\cdot A_{i}+f\\ \;\tau \dot{y_{i}}=z_{i}\\ f(W_{i},p)=\frac{\sum_{j=1}^{N}\psi_{j}v_{j}}{\sum_{j=1}^{N}\psi_{j}}p_{i} \end{array}$$

(2) $$\begin{array}{l} \;\psi_{j}=exp\left(h_{j}(cos(W_{i}-c_{j})-1)\right)\\ \tau {\dot{g}}_{i}=\alpha_{g}(g_{0}-g_{i}) \end{array}$$

(3) $$\begin{array}{l} \alpha =8.0,\beta =\frac{\alpha }{4},\alpha_{g}=\frac{\alpha }{2}\\ c_{j}\sim (0,2\pi ) \end{array}$$

where *z_i_*, *y_i_* and *g_i_* are the variables of the motor primitive. *amp* is the weight of correspondent input from PF layer and set to 27 which makes the output of motor primitives oscillate between −1 and 1. *A_i_* is the “clocking” input from the baseline behavior (the PF layer). τ is the time constant which is equal to the period ($\frac{1}{\omega_{i}}$) of input *A_i_*. *f* is the forcing term in which ψ_*j*_ are fixed basis functions, *v_j_* are the weights and *p_i_* is the amplitude which is equal to *amp*. *N* = 50 represents the number of basis functions. Using nonlinear arbitrary functions in *f* is a well-defined approach in machine learning (Bishop, 2006) for nonlinear regression and analogous to population coding in computational neuroscience (Dayan, 2005). In Equation (2), *h_j_* is a constant equal to 2 · *N* and *W_i_* is the phase input from baseline behaviors. *c_j_* is a vector containing N separations of the scope in (0, 2π). *g*_0_ is the anchor point (*g*_0_ = 0). Equation group (3) guarantees the damping convergence of the DMPs.

Theoretically, the forcing term *f* above is used as a universal signal modifier. Assume *F* = amp· *A_i_* + *f* is equal to the second-order equation based on the optimal signal (*y*_*op*_):τ^2^*ÿ*_*op*_ + τα*ẏ*_*op*_ + αβ*y_op_* − αβ *g_i_*, then substitute this equation to replace the *F* term, then we can rewrite the DMPs equation:

(4) $$\dot{z_{i}}=\tau {\ddot{y}}_{op}+\frac{\alpha }{\tau }\left(\tau {\dot{y}}_{op}-z_{i}\right)+\frac{\alpha \beta }{\tau }(y_{op}-y_{i}),\tau \dot{y_{i}}=z_{i}$$

According to the theory of morphed oscillation (Ajallooeian et al., 2013), Equation 4 is a second order morphed oscillator which can adapt the baseline behavior into any limit cycle shape. Then the task left is to figure out a mechanism for changing the parameters of DMPs to converge to a desired gait. Therefore, the DMP model is used as an interface for learning/adaptation.

In fact, DMPs are widely used to model discrete motor learning (Peters, 2007; Kober et al., 2012) and rhythmic movement (Ijspeert et al., 2013). In terms of periodic movement learning, Nakanishi et al. (2004) and Gams et al. (2009) employed demonstrated signals to learn motor primitives of rhythmic motion with local weighted regression. However, supervised learning might not always be the case for locomotion learning. Infants learn to crawl by interacting with the environment rather than being demonstrated how each joint moves dynamically (Clearfield, 2004; Kail and Cavanaugh, 2012). Locomotion learning based on reinforcement learning (RL) without demonstrated signals and motor primitives is also popular (Morimoto et al., 2005; Endo et al., 2008). However, as the motor primitives model has a good learnability, in this article, an approach of using motor primitives and RL for locomotion learning without demonstrated signals is proposed.

### 2.2. The mechanism: CPG-Actor-Critic

The CPG-Actor-Citic architecture has been used for exploring and learning complex locomotion patterns for both bipeds (Nakamura et al., 2007; Endo et al., 2008; Li et al., 2013b) and quadrupeds (Kohl and Stone, 2004; Li et al., 2013a). Inspired by Grillner et al. (2008), the functions of CPG-Actor-Critic connects the layered architecture to an behavioral selection learning (RL) process in which the optimal parameters of CPGs are determined. The actor, by generating actions, explores the state space of the body and the critic evaluates the actions taken by observing rewards in order to send improved control signals to the body. In this article, two modern policy-focused RL techniques are used for this continuous-space learning problem of DMPs. One is episodic natural actor critic (eNAC) (Peters, 2007) and the other is policy learning by weighting exploration with returns (PoWER) (Kober et al., 2012). eNAC has been empirically demonstrated to be a “winner” algorithm for policy gradient approaches, outperforming FDG (Finite Definite Gradient) and VPG (“Vanilla” Policy Gradient) (Peters, 2007). Also, PoWER is a faster RL learning algorithm better than the other “family” members, such as eRWR (episodic Reward-Weighted Regression) (Kober et al., 2012). In this article, we also intend to implement and compare these two state-of-the-art algorithms on locomotion learning tasks using DMPs.

Generally speaking, in the parameter space for using DMPs, a policy-based actor is used to sample/explore the continuous action space. Equation 5 shows a time-variant actor used for DMPs.

(5) $$\begin{array}{l} \;a=\theta^{T}\psi_{f}(x,t)+\epsilon_{t}\\ \epsilon_{t}^{n}\sim N\left(0,{\left(\sigma^{n}\right)}^{2}\right) \end{array}$$

where *a* is the output vector of an actor and **θ** is the policy parameter vector reflecting the weights *v_j_* in motor primitives. **ψ_*f*_** is the vector of normalized basis functions of motor primitives. **ϵ_*t*_** is the gaussian exploration vector with standard deviation **σ** which contains the standard deviation for each basis function at time *t* and ϵ*^n^_t_* is the exploration for the *n*th basis function in **ϵ_*t*_**. In most of cases, the **σ** is kept constant for all the parameters. It has been mathematically proved that both eNAC and PoWER using DMPs can also use an actor exploring only in the state-dependent parameter space (Shown in Equation 6) (Kober et al., 2012).

(6) $$\begin{array}{l} \;a=(\theta^{T}+\epsilon_{t}{}^{T})\psi_{f}(x,t),\;t=1,2,3...T\\ \epsilon_{t}^{n}\sim N(0,\;{\left(\sigma^{n}\right)}^{2}) \end{array}$$

According to Kober et al. (2012), the disadvantages of using state-independent exploration is: (1) Large variance in parameter updates. (2) The effect of perturbation could be washed out if exploration is too frequent as the system works like a low-pass filter. (3) It could possibly damage the completeness of system execution. On the other hand, the advantage of using state-dependent exploration in the actor is able to reduce the computational load by not executing a matrix multiplication **θ^*T*^_*t*_ψ_*f*_(*s, t*)**. Moreover, when the DMPs are used in the case that each basis function is activated only once in one period, the exploration can be further simplified to be executed at the beginning by using a time-invariant exploration **ϵ**^*T*^**_0_**. In the following section part, the update mechanism of the critic for each algorithm will be introduced.

#### 2.2.1. Policy gradient approach

Policy gradient is a well-established method used to update the parameterized action space. Since the normal “vanilla” gradient suffers the slow learning rate, natural policy gradient is formed by adding a regularized term in normal gradient approach to force the update path to follow the steepest direction (Kober et al., 2012).

Since learning locomotion might be a repetitive task (Adolph et al., 2012), episodic natural actor critic (eNAC) using natural gradient is selected. NAC is proposed by Kakade (2001) and further developed and used in motor learning by Peters (2007); Peters and Schaal (2008). eNAC is mathematically constructed on the NAC approach and uses episodic exploration for each rollout. In the practical work, eNAC generates a sufficient number of rollouts in order to get the realization of gradient information around the current state. It transforms the traditional RL problem of solving the Bellman equation to an explorative process of linear regression using DMP basis functions. Assume there are H rollouts the eNAC algorithm generates for each update, then the update rule for the critic can be summarized as below (for a mathematical introduction to eNAC, please refer to Supplementary Material):

$$\begin{array}{l} \left[\begin{array}{c} w\\ J \end{array}\right]={\left(\phi \phi^{T}\right)}^{-1}\phi R.\\ \;\phi ={\left[\sum_{t=1}^{s}\alpha_{t}▽log^{T}(\pi^{\theta }(u_{t}|x_{t}))w,1\right]}_{1:H}^{T}\\ \;R={\left[\sum_{t=1}^{T}\alpha_{t}r(x_{t},u_{t})\right]}_{1:H}^{T}\\ \;\theta^{\prime }=\theta +\alpha w \end{array}$$

where 1 : *H* represents H samplings within one trial (refer to details in the Algorithm). **ϕ** is the basis matrix containing H basis vectors for H rollouts and constant 1 in it is used to determine the baseline *J* avoiding large-variance updates. α_*t*_ is the theoretical discounting factor. ***R*** is the average reward vector in which *r* is the instant reward (for the detailed eNAC proof, please refer to Peters, 2007). ***w*** is the estimated steepest gradient according to your sampling rollouts and used to update the parameter **θ** to **θ′** with α learning rate (α = 0.1).

#### 2.2.2. Expectation Maximization based policy search

Expectation maximization (EM) is a useful tool as a machine learning technique to find out the optimal solutions based on increasing the value of the lower bound for the cost function (Bishop, 2006). According to Kober et al. (2012), a critical drawback of policy-gradient is its difficulty in determining the learning rate and the unsteadiness to reward values. This is why usually EM-based policy approaches can converge faster than policy-gradient approaches. A normal EM-based policy search algorithm works to find out the **θ′** which maximizes the lower bound *L*(θ′) on a cost function. Likewise, in order to find the maximum value of *L*(**θ′**), the derivative of it is set to zero (Equation 7, for the details please refer to Supplementary Material).

(7) $$\partial_{\theta^{\prime }}L(\theta^{\prime })=0$$

With the actor in Equation 5, it can generate an algorithm called episodic reward weighted regression (eRWR). But in our work, authors are more interested in a more efficient algorithm derived from the actor using the Equation 6, the policy learning based on weighted exploration with returns (PoWER). The derivation of PoWER is given in Supplementary Material. The update rule of PoWER is:

$$\begin{array}{l} \theta^{\prime }=\theta +{\left(\sum_{t=1}^{T}W(s,t)Q^{\pi }(s,a,t)\right)}^{-1}\\ \;\left(\sum_{t=1}^{T}W(s,t)\epsilon_{t}Q^{\pi }(s,a,t)\right) \end{array}$$

Where ***W*(*s, t*)** = **ψ**(***s***, *t*)**ψ**(***s***, *t*)^*T*^ (**ψ**(***s***, *t*)^*T*^**Σψ**(***s***, *t*))^−1^. **ϵ_*t*_** is the exploration in Equation 6 and **Q**(***s***, ***a***, *t*) is the action-state function based on the policy π with exploration **Σ** which is a diagonal deviation matrix.

#### 2.2.3. Continuous action space learning logic

In the above-mentioned algorithms, the convergence condition is set to be **θ′** ≈ **θ** which might be difficult to achieve when the reward function is not explicitly bounded as in the case of supervised RL with clear targets (Peters, 2007). Therefore, a general continuous action space learning logic is required to help us intuitively judge if the algorithm converges or not. Cacla (continuous action learning automaton) proposed by van Hasselt and Wiering (2007) and proved to outperform some typical RL methods (like SARSA, Q(λ), NAC and so on) (Hasselt, 2007), offers an update logic by exploring around the current state. If the value of the state increases after the action is taken, the update is executed. The schema of Cacla is shown in Algorithm 2.2.3. In the case of episodic learning, Monte-Carlo difference is used instead of typical temporal difference since the prediction value function *V*(***s*′**) for next future step ***s*′** is not explicitly observable (Jaakkola et al., 1995).

Algorithm 2.2.3

- Initialize the parameter **θ** and state space ***s***
- Repeat:
  ° Perform the exploration on policy π(***a***|**θ**, ***s***) and generate actions for rollouts (the number of rollouts *H* > 1).
  ° Calculate the value function *V* difference between current state ***s*** and the future state ***s′***:
  ° Value function estimation/approximation:
    1. Temporal difference: δ = *r* −λ *V*(***s*′**) + *V*(***s***), estimate *V*(***s′***) = *V*(***s***) + βδ, where *r* is the immediate reward and β is the learning rate.
    2. Monte-Carlo difference for episodic learning: δ = *R* − *V*(***s***), estimate *V*(***s′***) = *V*(***s***) + βδ, where *R* is the reward for one episode.
  ° Update judge: if δ > 0: Update the policy toward the good actions with gradients.
- Until no update is executed as the algorithm cannot find any better solution any more.

Using Cacla as an update logic is very useful for determining the condition of update (δ > 0) the termination of the algorithm for unbounded reward functions. In order to adapt eNAC and PoWER into the Cacla logic, we can modify the algorithm as follows (only the detailed modification is shown below neglecting the unchanged part):

- **....**
- Repeat:
  ° M trials each of which includes 10 rollouts (H = 10), In each rollout, action is generated by ***a*** = (**θ**^*T*^ + **ϵ**^*T*^**_*t*_**) **ψ_*f*_** where **ϵ_*t*_** ~ *N*(0, σ^2^ * **I**) (σ = 0.1) for t = 1, 2, 3 … .s
  ° Calculate the value function *V* difference between current state ***s***…
  ° **….**
  ° Update judge: if δ > 0: *eNAC* —- Calculate the gradient *g*:
    $$\left[\begin{array}{c} w\\ J \end{array}\right]={\left(\phi \phi^{T}\right)}^{-1}\phi R.$$
    where ***R*** = [*r*_1_, *r*_2_, ··· *r_H_*]^*T*^ and ϕ = [Ψ_1_, Ψ_2_, ···, Ψ_*H*_]^*T*^. *r_i_* represents the accumulated reward in this episode $r_{i}=\sum_{t=1}^{s}reward_{t}$ and $\Psi_{i}=\sum_{t=1}^{s}\sigma^{2}(\epsilon_{t}\psi )\psi^{T}$. Then update **θ′** = **θ** + α***w***
    *PoWER* —- Calculate the update for each parameter θ_*i*_: ${\theta^{\prime }}_{i}=\theta_{i}+\frac{E\left\{\sum_{t\;=\;1}^{T}\sigma_{i}^{2}\epsilon_{i,t}Q^{\pi }\right\}}{\sum_{t\;=\;1}^{T}\sigma_{i}^{2}Q^{\pi }}$
    where *Q*^π^ is the action-state function and ϵ_*i,t*_ represents the output of an actor for parameter i at time t. σ is the variance of the policy.
- **….**

Generally speaking, in this section, a locomotion system with the theme “two systems (the baseline system and DMPs) and one mechanism” is introduced as a design methodology for CPG-Actor-Critic. The design of each system also might be flexibly replaceable by more sophisticated systems. For example, the baseline behavior can be a complicated coupled network of which each node stands for one advanced baseline motion generator (Buchli et al., 2006; Gams et al., 2009). In the next section, we will test this method on two different robotic platforms.

## 3. Experiments and analysis

In this section, two experiments are reported for two different purposes. Firstly, the experiment 1 is to test and verify the learnability of the DMPs based CPG-Actor-Critic by using eNAC only since DMPs are always used in the regression approach. In this experiment, standard crawling (Wikipedia, 2013) is learned on the NAO robot which does not have pressure sensors for crawling. Secondly, the whole system is transferred to the ghostdog robot, whose body is softer and more flexible than the NAO robot, to test the transferability of the system to distinct morphologies and compare the two algorithms, eNAC and PoWER, on the more challenging locomotion task (more likely to find differences in performance this way).

### 3.1. Experiment 1: learning to crawl

The objective of experiment 1 is to verify the capability of DMPs based CPG-Actor-Critic on limit-cycle reshaping and postural control. There are two sub-experiments: One is to test the learnability of the CPG architecture by using a generic “reshaping” mechanism [Equation (1) with the same targeted posture-the same spineline angle]. The other is to test if the generic motor primitives can also adjust the joint posture (shifting centers of limit cycles) under the condition that the posture control reward is set to two different targets.

**Figure 4:**
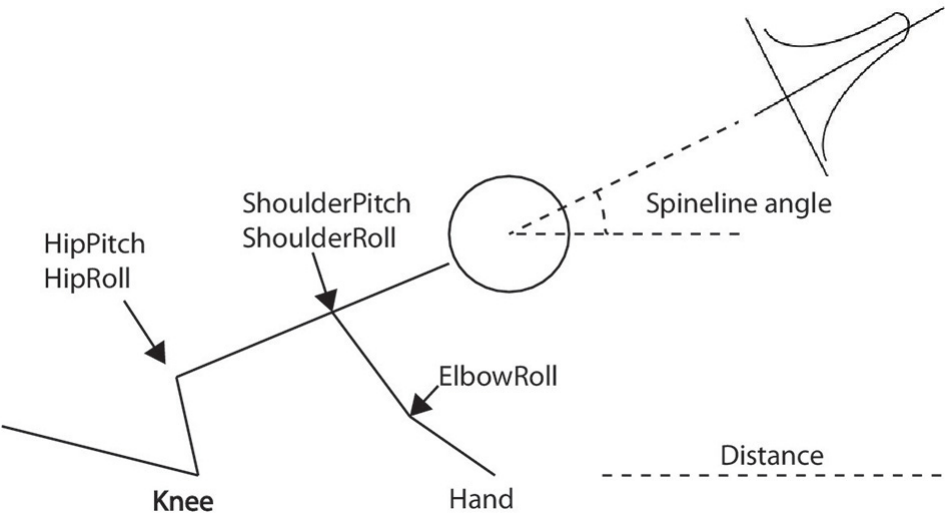
**The standard crawling posture on knees and hands and the main joints controlled by CPGs**. The distance and spineline angle indicate the quality of crawling. The spineline angle is controlled by a gaussian function.

With the eNAC algorithm above-mentioned, the robot is able to explore the dynamics of each joint on its own according to the specific reward function. Figure 4 shows the standard crawling (crawling on knees and hands Wikipedia, 2013). The main joints controlled by CPGs are the ones located at the hip and shoulder. The elbow oscillates with the rhythms of the shoulder pitch. Since crawling is left-and-right symmetric (Righetti et al., 2008), the number of degrees of freedom (DOFs) can be reduced from 8 (left and right joints) to 4 (left or right joints only). Therefore, the parameters for standard-crawling learning are 4·50 = 200 (where 4 is the number of DOFs and 50 is that of basis functions). From our previous work Li et al. (2013a), the move distance and spineline angle (Figure 4) are two significant factors to evaluate the quality of crawling behaviors. Accordingly, in the CPG-Actor-Critic architecture, the reward function is composed of two terms (*r_distance_* and *r_angle_*) as two evaluation landmarks for the above-mentioned two variables:

(8) $$\begin{array}{l} \;r_{reward}=r_{distance}+r_{angle}\\ r_{distance}=exp(\frac{D}{2})-1\\ \;r_{angle}=exp(e)-1\\ with\;e=N(x_{0},\sigma =0.02) \end{array}$$

where D is the distance the robot crawls every episode. e is a gaussian distribution with the center *x*_0_ and variance σ. Using *e* enables maintaining the posture of standard crawling without learning some extreme postures (Li et al., 2013a). In the case of infants learning to crawl, this function works similar to parents' hands adjusting or holding up the infant's body when (s)he is crawling.

#### 3.1.1. Sub-experiment: Reshaping-mechanism test

**Figure 5:**
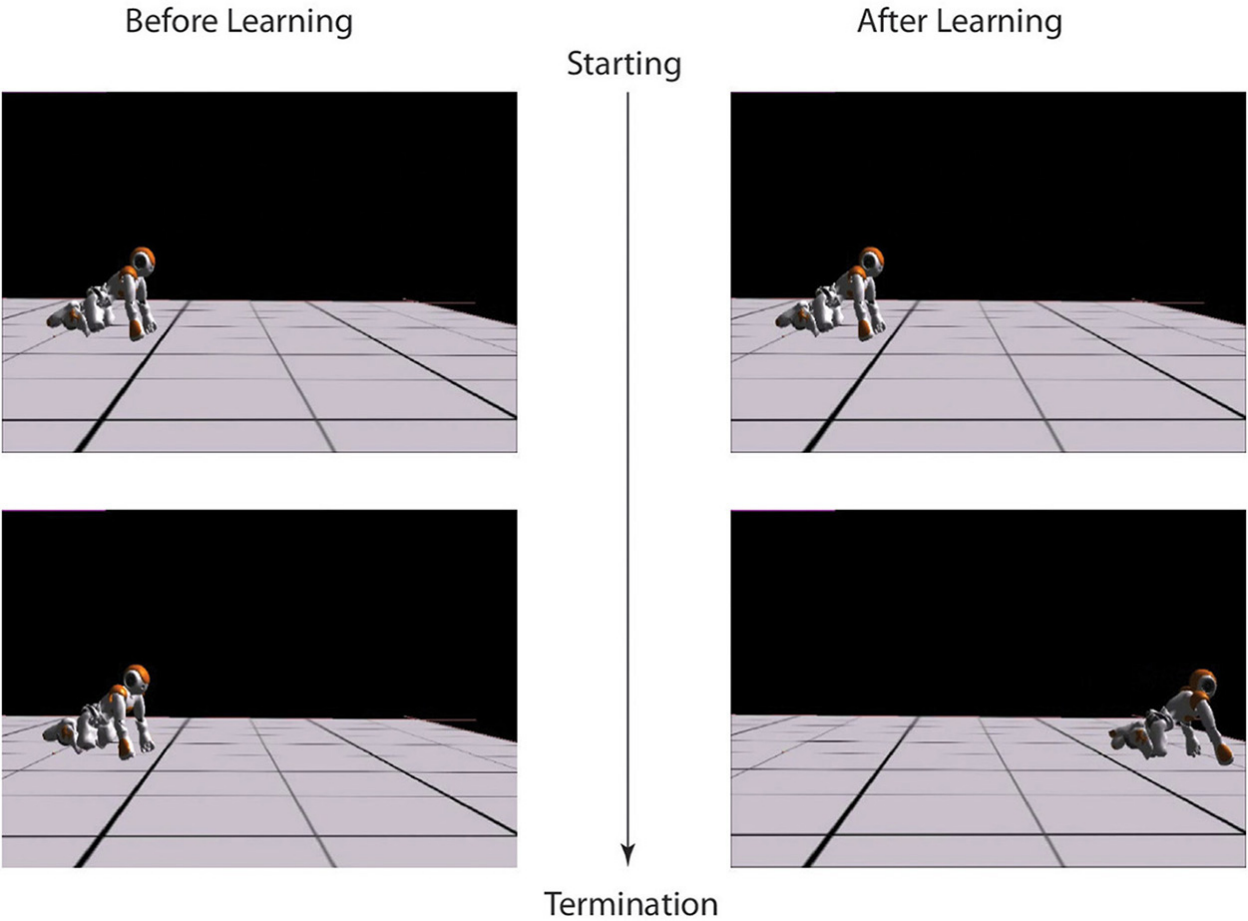
**Left:** the beginning and termination snapshots of crawling before learning. **Right**: the beginning and termination snapshots of crawling after learning.

**Figure 6:**
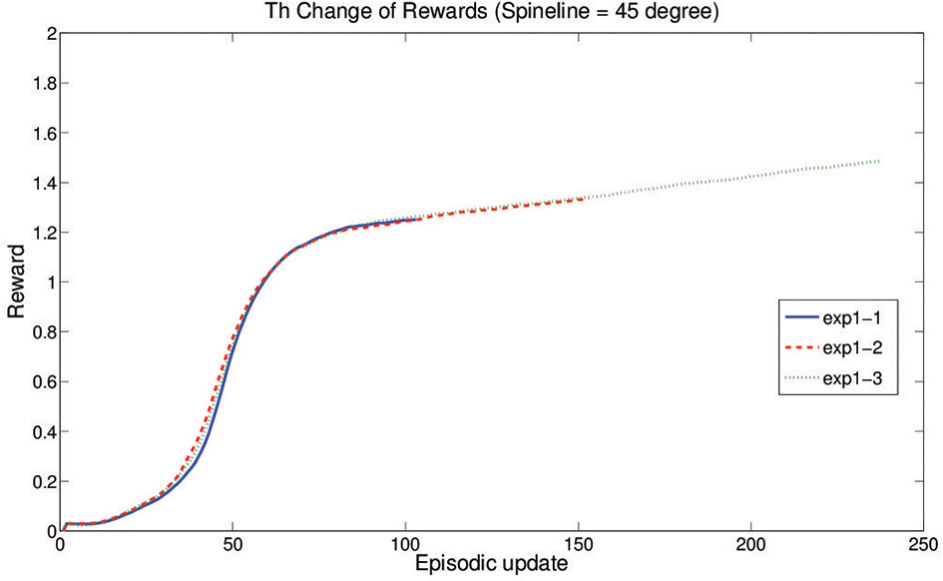
**Learning curves of three experiments**. exp1-n represents the result of the nth run in the experiment 1.

**Figure 7:**
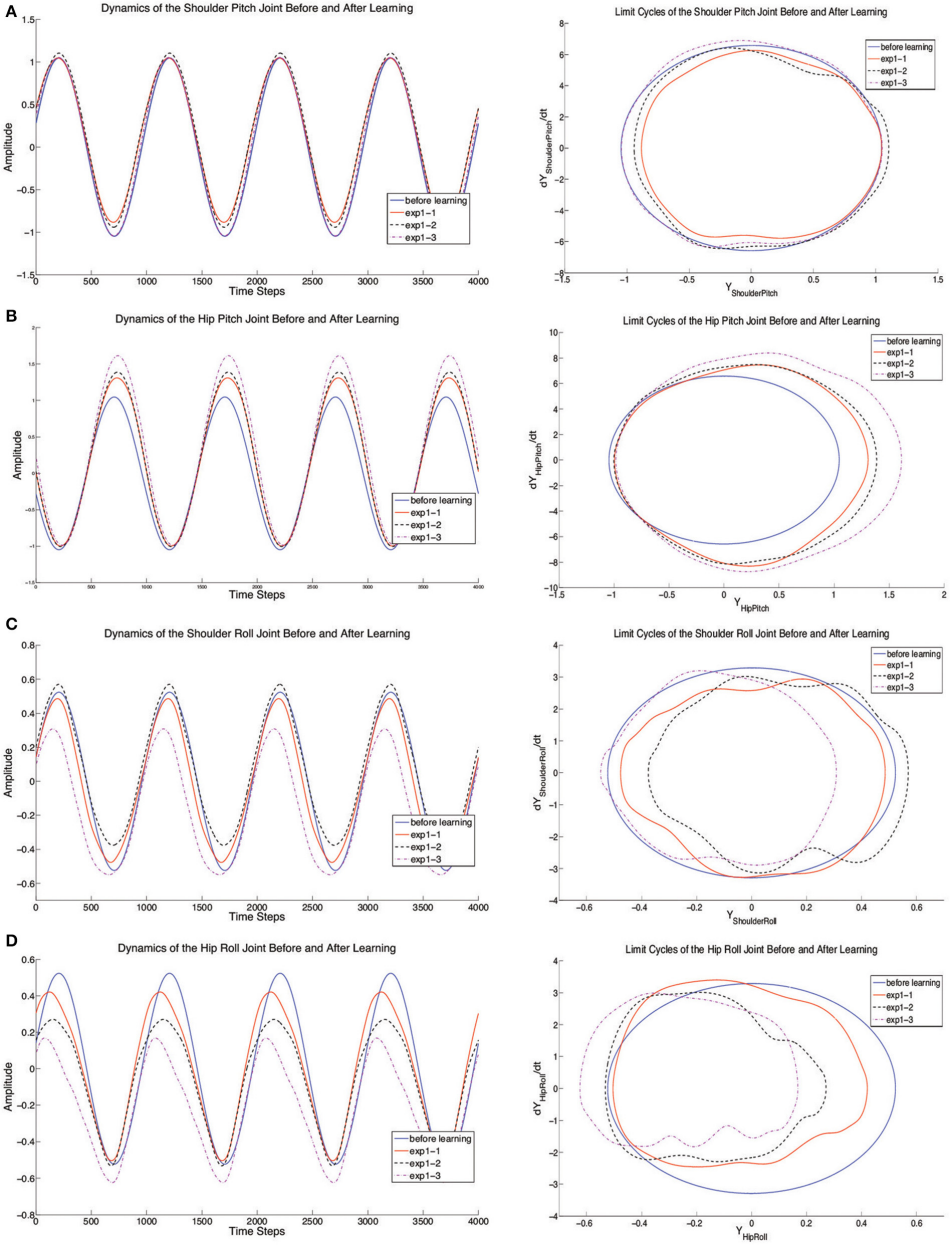
**The dynamics of joints (Shoulder Pitch and Roll, Hip Pitch, and Roll) before and after learning**. The left side of **(A–D)** represent the trajectory change for each joint and the right side of **(A–D)** represent limit cycles of each joint. In each figure, the blue line indicates the original joint motion without learning and the red solid lines, black dashed lines and purple dashed lines show the results of experiment 1–3 after learning.

In this Experiment, with the average spineline angle fixed at 30° (*x*_0_ = 1.05), the robot learns to crawl in three independent runs and finally converges to three different results by balancing the distance maximization and posture maintenance. Every experiment starts with the same initial posture with (*x*_0_ = 1.08, approximately 28°) and performs a pre-learning non-crawling behavior with no crawling distance (Figure 5). However, after learning, the standard crawling emerges through the interaction amongst the CPG-Actor-Critic architecture, the humanoid body and the environment (For the detailed performance, please refer to the video Li, 2013a). Interestingly, the three learning trials converge with similar smooth reward curves (Figure 6) but different results (Figure 7).

In order to clearly investigate the reasons of the formation of crawling, the joint dynamics are shown separately in Figure 7. Since the standard crawling is a whole-body motion, the CPG-Actor-Critic autonomously decides how to adapt the motion of each joint. The adaptive changes of pitch joints for shoulders and hips focus on the adjustment of their amplitudes (Figures 7A,B). Especially, the HipPitch joint tends to swing more backward so that robot can crawl forward with more force. Interestingly, extracting from the results in our experiments (Figures 7C,D), the significant factor determining whether the robot can crawl forward properly is the roll motion. Not only are the amplitudes of roll joints in shoulders and hips statistically adapted, but also are the phases of CPGs controlling roll joints shifted compared to the original CPG output without learning. It seems the DA layer modeled as motor primitives has the capability to deal with the reshaping of different limit cycle output and even to adjust the phase difference which is set inappropriately in the PF layer. Apart from these, it can also tune the posture. From the joint dynamics of roll motion, it is clearly observed that the limit cycles of roll joints are shifted, in which case the oscillation centers of roll joints are adaptively adjusted. Compared to the explicit posture-control terms in our previous work Li et al. (2013a,b), the implicit terms grounded in the motor primitives can integrate two functions: DA and posture control. To verify the functionality of posture control, we conduct a second experiment.

#### 3.1.2. Sub-experiment: Posture adjustment

In the experiment 2, the objective is to verify the capability of the proposed CPG-Actor-Critic architecture on the adjustment of joint posture. Actually, the spineline angle reward proffers a control signal of limiting the whole-body posture. With a loose control coefficient [e.g., σ > 0.02 in Equation (10)] or without the spineline restriction, the robot will only consider the maximization of crawling distance, ignoring the maintenance of the posture. This causes a convergence to an extreme crawling behavior. In human reality, parents always need to guide a right posture by holding up or lifting the infant's body when they are crawling. Therefore, the posture limitation is necessary.

**Figure 8:**
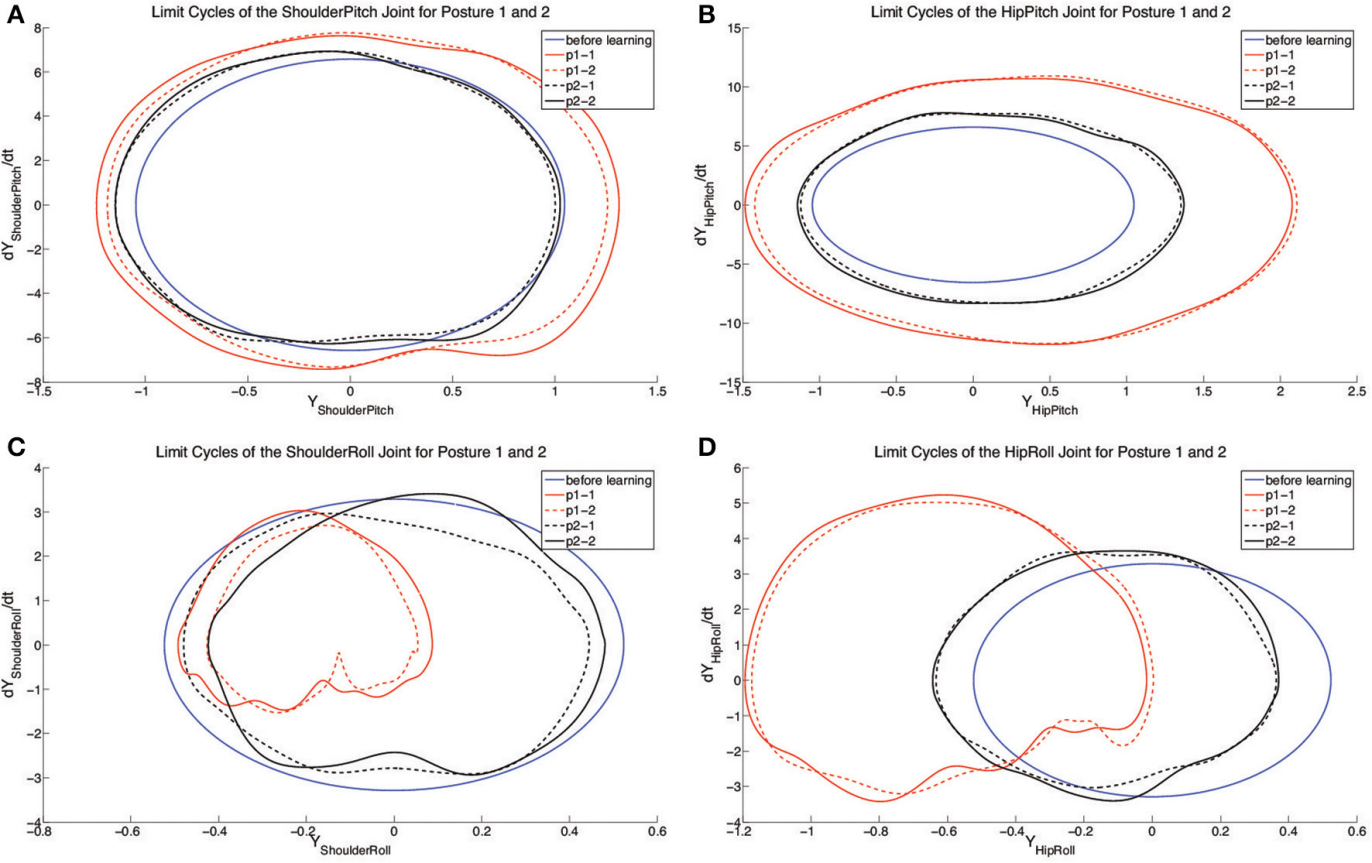
**The figure shows the limit cycles of each controlled joint with 2 independent learning experiments for two different body postures. (A–D)** represent the limit cycles of each joint before and after learning. The blue solid lines represent the limit cycles for each joint before learning. Red solid and dashed lines indicate the limit cycles for tested posture 1 (*x*_0_ = 1.03, ~31°). Black solid and dashed lines indicate the limit cycles for tested posture 2 (*x*_0_ = 1.08, ~28°). pn-n is the abbreviation of posture n-experiment n.

For testing the posture control abilities, two spinelines are chosen (*x*_0_ = 1.03 and *x*_0_ = 1.08, approximately 31° and 28°). Two independent learning experiments are performed respectively for each of these two spineline-angle controlled postures. With the results obtained, the comparison of limit cycles of joints in 4 runs are given in Figure 8. For each group of the results (black and red curves), the crawling joint dynamics converge to similar limit cycles. In terms of the motion of pitch joints (shoulders and hips), from Figures 8A,B, the deviation between two limit-cycle centers is blurry. However, it is conspicuous for the roll joints, especially hip roll joints (Figures 8C,D). The limit-cycle centers are both shifted rightwards for shoulder and hip roll motion from posture 2 (28° spineline angle) to posture 1(31° spineline angle). This limit-cycle-center shifts correspond to the closing-inward and opening-outward posture changes of shoulder and hip joints. This is a typical whole-body motion of lifting the center of gravity of the body and increasing the spineline angle. Compared to the explicitly allocated posture-change terms in previous work Li et al. (2013a,b), using motor primitives can interactively rule out the unnecessary joints for posture control. For example, in the experiment 2, to change from posture 1 to posture 2, the system determines to fixating on altering the posture of roll joints other than pitch joints based on the whole-body motion logic.

### 3.2. Transferred test on the physical robot

**Figure 9:**
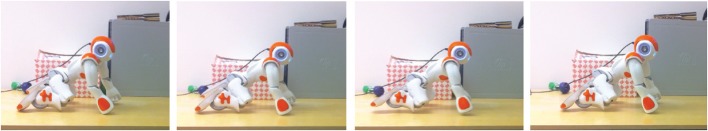
**The implementation on the physical robot**. This figure shows the video snapshot of one-crawling-step NAO robot on a wooden flat table (One crawling step means one time alternation of the supporting leg and arm).

In this article, with 7 learned sets of parameters, the learned CPGs are transferred to the physical NAO robot for testing. In all the experiments, the popular Webots simulator (Michel, 2004) based on ODE (open dynamics engine) is used. In order to successfully test the learned motion from the simulated robot to the physical one, some preconditions have to be realized. As discussed in previous work Li et al. (2013a), the possible failures of transferred results on physical robots could be caused by the disparity in physics engines and difference between simulation time and real time. In our work, the frequency of the CPG is doubled while being transferred. 5 out of 7 results can be successfully transferred except the results for the posture (*x*_0_ = 1.03). After the CPG amplitudes of pitch joints are reduced to 70%, the failed transferring becomes successful. Figure 9 shows the snapshots of one-step crawling on the physical robot (for details, please refer to the video Li, 2013b). Compared to the previous implementation with only optimized postures, the left-right curvy motion of the spineline, a typical characteristic of crawling behaviors (Righetti et al., 2008), emerges after learning.

### 3.3. Experiment 2: learning double-suspension gallop

In this experiment, the CPG-Actor-Critic architecture is transferred to the puppy robot (ghostdog) with a flexible body for learning the double-suspension gallop, a typical dog running Gait (dog). The rear limbs are synchronized in phase and so are the front limbs. The main joints controlled by the CPGs are the rear and front hip joints. The knee part of this robot is fully passive with a spring-damper system. In order to maximize the running distance, a reward based on the distance only is used, in which case the robot is required to run as fast as possible in a time-fixed episode. Similar to the reward function of learning to crawl, we adopt the distance-related part of that reward function as the only learning landmark. With both eNAC and PoWER, the objectives of experiment 2 include: 1. Test the morphological transferability of the CPG-Actor-Critic. 2. Compare the learning efficiency of eNAC and PoWER.

#### 3.3.1. Morphological Transferability

**Figure 10:**
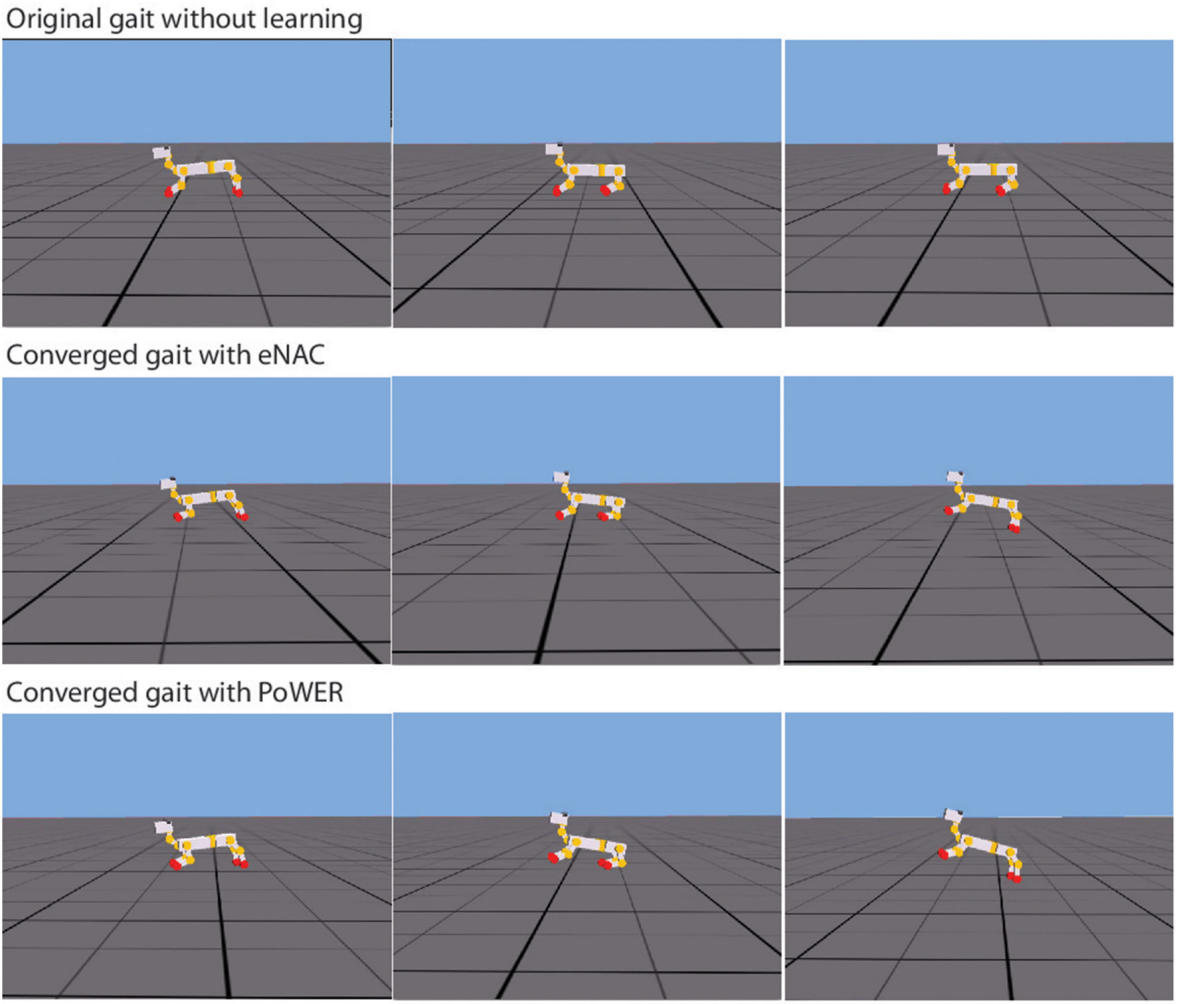
**The snapshots of gallop gait under three conditions**. The first row shows the original gait without learning, the middle row shows the converged gait after eNAC learning and the final row shows the converged gait after PoWER learning.

In the experiment 1, the learning architecture has already been demonstrated to be workable on a rigid-body system, the NAO robot. However, a complete architecture should also be able to work on different morphologies, especially in the case that a robot's body cannot be accurately modeled in terms of its morphological flexibility. Then the learning/adaptation system can help the robot to find out solutions by itself with some prior knowledge. In this article, the baseline behaviors serve as prior knowledge and the emergence of a particular gait based on this prior knowledge becomes so intriguing. Figure 10 presents the snapshots of double-suspension gallop gait before and after learning. The difference before and after learning is so conspicuous (For the details, please refer to the video Li, 2013c). Before learning, the puppy robot can move very hard by scratching the ground. But after learning, a new gait emerges from the previous ground-scratching behavior. In the new gait, it seems the robot can take advantage of its own characteristics of the physical body to move as fast as possible. In Figure 10, the third snapshot of two after-learning gaits both demonstrate that the robot uses the spring of rear legs to bounce up the whole body in the air so that it can move much further and the first snapshot of two after-learning gaits also both demonstrate that the spring of front legs are used to reduce the impact when the body hits the ground from the air. These phenomena possibly indicate that the CPG-Actor-Critic might have the ability to realize the morphological advantages of a certain body.

**Figure 11:**
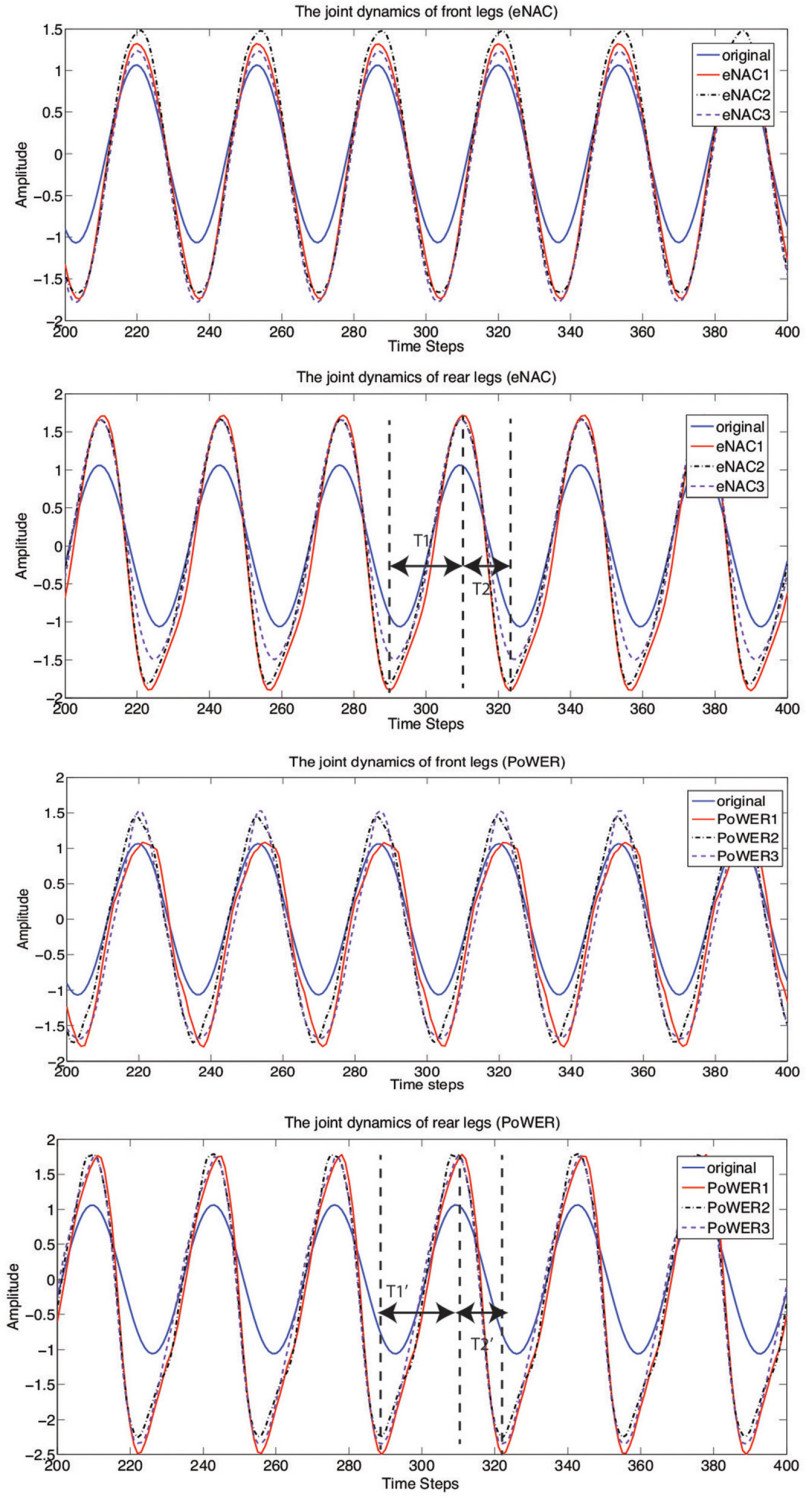
**The front and rear joint dynamics of the ghostdog robot before and after learning with eNAC and PoWER in three trials (marked by eNAC1~3 and PoWER1~3)**. *T*1, *T*2, *T*1′, and *T*2′ represent the ascending time and descending time of the joint dynamics tuned by eNAC and PoWER after learning. Before learning, *T*1 = *T*2 and *T*1′ = *T*2′.

In details, Figure 11 presents the learning results of converged joint dynamics. For each RL technique of eNAC and PoWER, there are three learning trials conducted with the same initial conditions (posture and position). The ghostdog robot learns repeatedly in the simulator and is automatically reset by supervisor functions according to Michel (2004). It is shown in Figure 11 that each batch of three trials by eNAC and PoWER qualitatively converge to similar joint dynamics respectively. There are some common features of the converged dynamics captured by both eNAC and PoWER. Firstly, as for the front legs, the changes focus on increasing the amplitude of joint oscillation. Secondly, as for the rear legs, both eNAC and PoWER change the originally equal stance and swing phase to the dynamics in which the stance phase is much longer than swing phase. In terms of double-suspension gallop, a longer stance phase (*T*1 and *T*1′) on the rear legs drive the front legs off the ground and a shorter swing phase (*T*2 and *T*2′) of the rear legs makes them follow the move direction and finish the phase transition from swing to stance as fast as possible when the front legs hit the ground.

#### 3.3.2. Comparison between eNAC and PoWER

**Figure 12:**
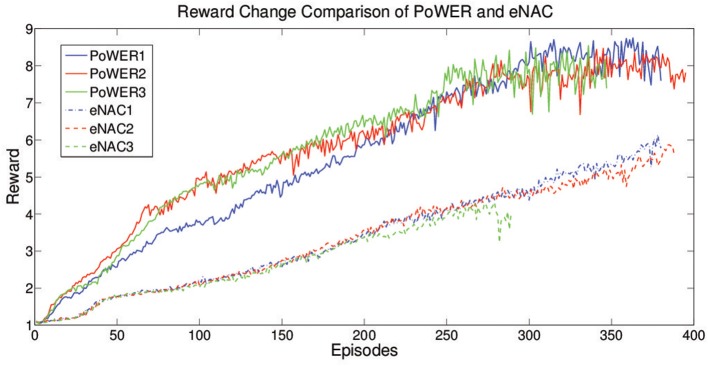
**The learning curve of eNAC and PoWER over 400 episodes which comprise three trials**. The reward reflects the distance the robot moves in the same period.

According to Kober et al. (2012), as a typical gradient based approach, eNAC suffers the problem of finding out a proper learning rate in the supervised learning. It is an open problem for policy gradient approaches in terms of good learning performance but EM-based policy search can avoid this problem. In order to compare eNAC and PoWER for RL cases, a stable learning rate is chosen to be used in eNAC (α = 0.1, when α > 0.5, five trials are conducted but none of them can successfully and stably learn with more than 20 updates (less than 20 updates is considered as failure). When α = 0.2, 0.3, 0.4, the failure rate is about 20%, 20%, 40% for five trials respectively). Figure 12 shows that PoWER outperforms eNAC in learning speed by boosting the reward in three trials respectively. eNAC is able to optimize the moving distance but it gets stuck in some local optima while PoWER can find better solution (further distance in the same period). This result is similar to the cases presented in Kober et al. (2012) supervised learning experiments (i.e., PoWER outperforms eNAC in terms of learning rate and results). Nevertheless, eNAC is very sensitive to the reward value. When the reward value increases, the update variance starts to increase and the stability of learning starts to deteriorate. But PoWER is quite stable to the reward value change. In terms of convergence, the convergence of PoWER and eNAC is determined by δ with Cacla logic. Because of the different learning speed, Cacla converged as no further expected return of samples can make δ positive so that the final converged expected reward oscillates around the average optima (Figure 12). However, eNAC converged as δ is almost zero (|δ| < 10^−4^).

### 3.4. Summary

In this section, the DMPs based CPG-Actor-Critic architecture is tested for its learnability on a rigid body robot, the NAO humanoid, for learning to crawl. Then it is transferred to a “soft”-bodied robot, the ghostdog robot, for learning double-suspension gallop. Two advanced RL algorithms (eNAC and PoWER) are utilized and the results are compared. With two state-of-the-art RL techniques, qualitatively similar gaits emerge from the interaction amongst the CPG controller, the environment and the body in an actor-critic mechanism. In both experiments, DMPs work as an universal limit cycle modifier to reshape the existing baseline behavior into optimal gait dynamics. The functionality of DMPs is a mathematical framework for optimizing/learning locomotion gaits without explicit sensory feedback but serves as a mechanism functionally similar to sensory-feedback-reshaping (Grillner et al., 2008; Ijspeert et al., 2013).

## 4. Conclusion

### 4.1. eNAC and PoWER

eNAC and PoWER, as two different RL algorithms, are based on the policy gradient and the EM-based policy search, respectively. They are compared the first time for learning a periodic motion. According to the results in Experiment 2, we can summarize the difference between them as follows:

1. Learning speed. PoWER searches a better policy faster than eNAC as the small learning rate α of policy gradient slows down the learning speed of policy gradient approaches.
2. Converged results. Both PoWER and eNAC possibly get stuck in local optima according to Kober et al. (2012). However, PoWER can converge to better results than eNAC.

### 4.2. DMPs based CPG-Actor-Critic

In this article, the proposed CPG-Actor-Critic based on DMPs seems to be able to optimize/learn a gait given an initially rough baseline behavior and a body. The forcing term *f*(*W_i_, p*) in Equation (1) works like sampling sensors which “perceive” a sufficiently large number of proprioceptive points of the CPGs so that it can adapt them flexibly into distinct dynamic patterns on the basis of actor-critic interaction. Even though this implementation of motor primitives with RL approaches instead of supervised learning opens a new page for locomotion learning, this approach still has some disadvantages: Firstly, learning might be slow. In the experiments, each one takes about 6–7 h to complete. Therefore, the learning process might not be transferred to the physical robot. On the other hand, a fast learning mechanism might be needed for a fast adaptation to the dynamical environmental changes in our architecture. The potential solution is either to develop a higher level of cognitive interpretation of environmental needs for switching different learned gaits (e.g., in Aoi and Tsuchiya, 2005) or use a faster learning algorithm in a fast adaptation mechanism (e.g., in Manoonpong et al., 2013). Secondly, the frequency is not adaptive. In the work presented above, all the CPG frequencies are fixed. Even though the motor primitives can innately preserve the learned dynamics when the frequency is changed, it still cannot guarantee that the new frequency patterns still can work when the whole-body dynamics change with the oscillation frequency. As a matter of fact, after reducing the frequency from 1.0 to 0.5, the robot's body dynamics change and the crawling in experiment 1 cannot be properly presented. The solution to this problem might be to use hybrid learning based on eNAC by counting in the frequency parameters (Kober, 2012). Thirdly, the implementation of CPGs is not energy efficient. In our work, the CPGs are used as trajectory generators. The layered architecture still lacks an adaptive approach to altering the stiffness of joints. On the other hand, low energy efficiency might be a natural flaw of rigid body robots. Even though force control might be able to improve the energy issues on a rigid body, the inflexibility of the body is still a stumbling block preventing a robot from being energy-efficient for locomotion. Finally, the lack of a memory architecture. Even though DMPs can optimize/learn gaits from the prior knowledge, it cannot memorize the relation between the environment and parameter space. This is an open problem for locomotion modeling.

In conclusion, DMPs based CPGs are able to not only learn demonstrated/supervised signals (Gams et al., 2009) but also adapt to flexible patterns based RL approaches in our work. Even though it is not a complete solution for the integration of sensory feedback (e.g., moving distance, spineline angle and muscle reflex. Muscle reflex is not used in our work since the NAO robot does not have pressure sensors on the hand), it offers a mathematical solution to mimic the same function of sensory feedback in reshaping and shifting limit cycles. The advantage of using DMPs as a dynamic adaptation tool can be summarized as follows:

1. *an optimizer for an existing gait*. Since there is no context-free locomotion capability and the environment is not possible to model, using a well-designed gait and optimize it in the environment in which the original gait cannot work well.
2. *a gait searcher*. Given prior knowledge about a certain gait and a body, locomotion modelers might have difficulty in determining the detailed motion of each DOF/joint. Using DMPs with RL mechanism enables seeking out the optimal solution. In this article, two experiments show the process of the emergence of a particular gait based on its baseline motion (prior knowledge).

### 4.3. A generic view: two systems and one mechanism

A lot of inspiration related to locomotion learning/development can be extracted from cognitive science (e.g., Thelen, 1996), neuroscience (e.g., Schore, 1994; Grillner et al., 2008), psychology (e.g., Clearfield, 2004; Adolph et al., 2012) and robotics (e.g., Pfeifer and Bongard, 2006). From the perspective of Thelen (1996), locomotion development/learning is focused on the formation and adaptation of the so-called “attractors” in a dynamic system. This assumption indicates that locomotor system design should not be focused on how a static system can be modeled but how a dynamic system might develop in the interaction with environments. The stagnation is only one “special” attractor of the system. In this sense, DMPs have been assumed to represent locomotion attractors in Ijspeert et al's work (Ijspeert et al., 2013). Both Schore (1994) and Grillner et al. (2008) imply that locomotion learning might be RL-related from the perspective of neuropsychology and neural structures. From the psychological point of view, Clearfield (2004) indicates the developmental relation of locomotion to spatial memory including the distance. Adolph et al. (2012) recently explained why the repetitiveness is important for infants to learn locomotion. Finally, Pfeifer and Bongard (2006) rethink the locomotion and emphasize the interaction between the body and the environment. Based on the above view of locomotion from different angles, locomotion learning is an affective-related, interactive and repetitive process with cognitive cues.

Therefore, in order to have a sketch of a dynamic locomotor system composed of the three components mentioned in Section 1, in this article, we propose the “two systems and one mechanism” architecture. Two systems cover one baseline motion system and one adaptation system. The former includes a general model which is able to handle basic locomotion functions (e.g., the coordination and synchronization of DOFs, gait transition). The latter comprises basic adaptation function/interface to adapt basic locomotion dynamics into context-specific dynamics. One mechanism is an affective-modulated process of organizing how the DA can happen with more complex perceptual information (e.g., visual signals) and determining what context-specific dynamics the locomotor system should adapt into.

**Figure 13:**
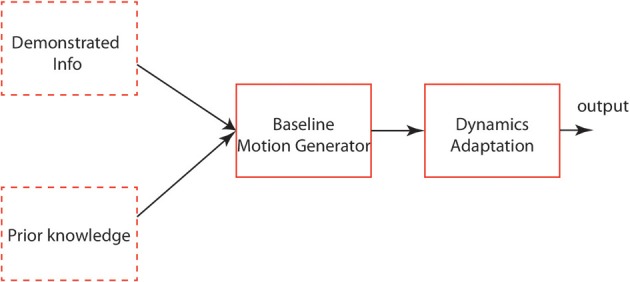
**The generic architecture of locomotion learning**. The dashed-line blocks represent the choices of input. The other blocks represent the functions of each layer.

The general architecture based on the points above-mentioned in Figure 13 explains an applicable schema for locomotion learning. The baseline motion generator can be modeled based on demonstrated signals (Nakanishi et al., 2004; Gams et al., 2009) or prior knowledge (Righetti, 2008). After this is accomplished, the basic motion dynamics is to be adapted into context-related dynamics by an interactive mechanism. In our modeling approach, the four-cell architecture encodes the basic patterns of different kinds of gaits. Each cell in this architecture can also be modeled as a complicated neural system and more neural systems are coupled in this network. DMPs, as a mathematical dynamics modifier, work with actor-critic RL mechanism (Kober and Peters, 2010) to optimize/learn a locomotor system. In the future work, a sophisticated memory system, which includes short-term memory and long-term memory, is required in our system to map the contextual factors into parameter space. Also, a sensory feedback integration will be considered to be used together with DMPs based CPG-Actor-Critic.

### Conflict of interest statement

The authors declare that the research was conducted in the absence of any commercial or financial relationships that could be construed as a potential conflict of interest.

## Footnotes

^1^In Ijspeert et al's work, they refer to “dynamic movement primitives” which is “dynamic motor primitives” in this article.
