# Supplementary material for: A novel approach to locomotion learning: Actor-Critic architecture using central pattern generators and dynamic motor primitives
Source: Front Neurorobot. 2014 Oct 2;8:23. doi: 10.3389/fnbot.2014.00023 (PMC4183130; doi:10.3389/fnbot.2014.00023)
Supplement: Supplementary file 4 [file Presentation1.PDF]

# A Novel Approach to Locomotion Learning: Actor-Critic Architecture using Central Pattern Generators and Dynamic Motor Primitives

Cai Li[1], Robert Lowe[1], and and Tom Ziemke[1,2]

[1]Interaction Lab, School of Informatics, University of Skövde, Skövde, Sweden,
[2]Department of Computer and Information Science, Linköping University, Linköping, Sweden

## Appendix A
## Proof of cyclic $H/K$ for gallop gait

$H/K$ theorem:
"*Let $\Gamma$ be the symmetry group of a coupled cell network in which all cells are coupled and the internal dynamics of each cell is at least two-dimensional. Let $K \subset H \subset \Gamma$ be a pair of subgroups. Then there exist periodic solutions to some coupled cell systems with spatial-temporal symmetries H and spatial symmetries K if and only if $H/K$ is cyclic and K is an isotropy subgroup. Moreover, the system can be chosen so that the periodic solution is asymptotically stable.*"
Mathematically, $H$ is the group $\{x_1(t), x_2(t)\}$ with spatial temporal symmetry which means $x_1(t) = x_1(t+\theta) = x_2(t)$, where $x_1(t)$ and $x_2(t)$ are periodic signals, $\theta$ is the phase shift. For example, as the groups $((12),(34),\frac{T}{2})$ in the four-cell coupled network for the crawling gait, both cell 1,2 and cell 3,4 are symmetric with phase shift $\theta = \frac{T}{2}$. $K$ is the group $\{x_1(t), x_2(t)\}$ with spatial symmetry which means $x_1(t) = x_2(t)$. For example, as the groups $((14),(23),0)$ for the crawling gait, both cell 1,4 and cell 2,3 are equal to each other.
Assume a periodic signal $x(t) = x(t+T)$, so group $\{x(t), x(t+\frac{T}{2})\}$ is a cyclic group generated by $\frac{T}{2}$. The order of this group is 2 since $x(t+\frac{T}{2}+\frac{T}{2}) = x(t+T) = x(t)$, so it follows the $Z_2$ symmetry. Generally, assume the period $T$ is divided by m, then a group $Y: \{y_0, y_1, y_2, y_3, y_4, ...y_{m-1}\}$ where $y_0 = x(t), y_1 = x(t+\frac{T}{m}), y_2 = x(t+\frac{2T}{m}), y_3 = x(t+\frac{3T}{m})...y_{m-1} = x(t+\frac{(m-1)T}{m})$ and each signal in this group is phase shifted by $\frac{T}{m}$, so:

$$y_1 = x(t+\frac{T}{m}+\frac{T}{m}) = y_2$$
$$y_2 = x(t+\frac{2T}{m}+\frac{T}{m}) = y_3$$
$$y_3 = x(t+\frac{3T}{m}+\frac{T}{m}) = y_4$$
$$\cdots\cdot$$
$$y_{m-1} = x(t+\frac{(m-1)T}{m}+\frac{T}{m}) = x(t) = y_0$$

Therefore, if phase shift is done m times, the group will turn out to be original group. So group $Y$ is $Z_m$ cyclic. According to group theory[1], subgroups of cyclic group is also cyclic. Hence, any two of group Y form a group which is also cyclic. For example, group $\{y_0, y_i\}$ where $i < m$. If $m \gg 0$, the $y_i = x(t+\frac{iT}{m})$, always can find a value $a \in [0, T]$ and $\left|a - \frac{iT}{m}\right| \ll \epsilon$ where $\epsilon$ is a very small number. Therefore, $y_i$ can be $x(t+a)$ where $a$ is a value in [0, T], namely any group $\{x(t), x(t+a)\}$ is a cyclic group and $m \gg 2$. In our work, $a = 2$.

## Appendix B
## Mathematical Introduction to eNAC

Assume the stationary policy is $\pi^{\boldsymbol{\theta}}(\boldsymbol{x}, \boldsymbol{u})$ which can determine action space $\boldsymbol{u}$ based on state space $\boldsymbol{x}$ with a static distribution $d^{\pi}(\boldsymbol{x})$. The immediate reward is $r(\boldsymbol{x}, \boldsymbol{u})$. Then the expected reward $J(\boldsymbol{\theta})$ and normal policy gradient can be written as:

$$\bigtriangledown_{\theta} J(\boldsymbol{\theta}) = \int_{\boldsymbol{x}} d^{\pi}(\boldsymbol{x}) \int_{\boldsymbol{u}} \pi^{\boldsymbol{\theta}}(\boldsymbol{u}|\boldsymbol{x}) \bigtriangledown_{\theta} log(\pi^{\boldsymbol{\theta}}(\boldsymbol{u}|\boldsymbol{x})) \bigtriangledown_{\theta} log^{T}(\pi^{\boldsymbol{\theta}}(\boldsymbol{u}|\boldsymbol{x}))\boldsymbol{w} d\boldsymbol{x} d\boldsymbol{u} \quad (1)$$

$$\boldsymbol{\theta}_{n+1} = \boldsymbol{\theta}_n + \alpha \bigtriangledown_{\theta} J|_{\boldsymbol{\theta}=\boldsymbol{\theta}_n} \quad (2)$$

where the policy $\pi^{\boldsymbol{\theta}}(\boldsymbol{x}, \boldsymbol{u})$ is derivable at the policy parameters $\boldsymbol{\theta}$, namely $\bigtriangledown_{\theta}\pi^{\boldsymbol{\theta}}$ exists. For maximizing expected reward $J(\boldsymbol{\theta})$ with respect to $\boldsymbol{\theta}$, policy gradient will find the steepest increase direction $\bigtriangledown_{\theta} J = J(\boldsymbol{\theta} + \triangle\boldsymbol{\theta}) - J(\boldsymbol{\theta})$ to update searching policy $\pi^{\boldsymbol{\theta}}(\boldsymbol{x}, \boldsymbol{u})$ until it converges. n represents the nth step of update and $\alpha$ is the learning rate (equal to 0.01). By and large, Equation (4) and (5) plot the rudimentary rule of thumb for policy gradient approaches. By using natural policy gradient, the normal form is turned into:

$$\boldsymbol{\theta}_{n+1} = \boldsymbol{\theta}_n + \alpha F_{\theta}^{-1} \bigtriangledown_{\theta} J|_{\boldsymbol{\theta}=\boldsymbol{\theta}_n} = \boldsymbol{\theta}_n + \alpha \boldsymbol{w} \quad (3)$$
$$F_{\theta} = \int_{T} \pi^{\boldsymbol{\theta}} \bigtriangledown_{\boldsymbol{\theta}} log\pi^{\boldsymbol{\theta}} \bigtriangledown_{\boldsymbol{\theta}} log\pi^{\theta} d\boldsymbol{\theta}$$

where F is the Fisher Matrix (FM) and $\boldsymbol{w}$ is the weight vector. Multiplied by FM, normal policy gradient is changed to the steepest one. In equation (4), $\bigtriangledown_{\theta} log(\pi^{\boldsymbol{\theta}}(\boldsymbol{u}|\boldsymbol{x}))$ is the basis function vector related to state space $\boldsymbol{x}$. Then RL problem is transformed to figure out the approximation of $Q(\boldsymbol{x}, \boldsymbol{u})$ function with the basis functions by searching for a proper weight vector. According to eNAC, the weights, obtained by

least square learning, can be employed to update the policy parameters directly:

$$\begin{bmatrix} \boldsymbol{w} \\ J \end{bmatrix} = (\boldsymbol{\phi}\boldsymbol{\phi}^T)^{-1}\boldsymbol{\phi}\boldsymbol{R}.$$

$$\boldsymbol{\phi} = [\sum_{t=1}^{s} \alpha_t \bigtriangledown log^T(\pi^{\boldsymbol{\theta}}(\boldsymbol{u}_t|\boldsymbol{x}_t))\boldsymbol{w}, 1]^T_{1:H} \tag{4}$$

$$\boldsymbol{R} = [\sum_{t=1}^{T} \alpha_t r(\boldsymbol{x}_t, \boldsymbol{u}_t)]^T_{1:H} \tag{5}$$

where $1 : H$ represent H times samplings within one trial (refer to details in the Algorithm). $\boldsymbol{\phi}$ is the basis vector and constant 1 is used to determine the baseline $J$ avoiding large-variance update. $\alpha_t$ is the theoretical discounting factor. $\boldsymbol{R}$ is the average reward vector in which $r$ is the instant reward (for the detailed eNAC proof, please refer to[2]).

## Appendix C
## Mathematical introduction to PoWER

Assume the expected return $J$ with policy $\pi$ and parameters $\boldsymbol{\theta}$ is expressed as:

$$J(\boldsymbol{\theta}) = \int_T p_{\boldsymbol{\theta}}(\boldsymbol{\tau})R(\boldsymbol{\tau})d\boldsymbol{\tau}$$

where $T$ is the set of all possible paths. $\boldsymbol{\tau}$ indicates one rollout $[s_{1:T+1}, a_{1:T}]$ which is generated every trial or episode. $s$ is the state space $[s_1, s_2, ..., s_{T+1}]$ and $a$ is the action space $[a_1, a_2, ..., a_T]$. $p(\boldsymbol{\tau})$ is the probability of generating this path $\boldsymbol{\tau}$. $R(\boldsymbol{\tau})$ is the return of the path $\boldsymbol{\tau}$. According to Dayan and Hinton[3], the success of reward-weighted path distribution is to minimize the Kullback-Leibler divergence $D(p_{\boldsymbol{\theta}}(\boldsymbol{\tau})R(\boldsymbol{\tau})||p_{\boldsymbol{\theta}'}(\boldsymbol{\tau}))$ between the new path distribution $p_{\boldsymbol{\theta}'}(\boldsymbol{\tau})$ and the reward-weighted previous one $p_{\boldsymbol{\theta}}(\boldsymbol{\tau})R(\boldsymbol{\tau})$, where the Kullback-Leibler divergence $D(p(\boldsymbol{\tau})||q(\boldsymbol{\tau})) = \int p(\boldsymbol{\tau})log\frac{p(\boldsymbol{\tau})}{q(\boldsymbol{\tau})}$. So the logarithm of $J(\boldsymbol{\theta})$ can be written like according to Jesen's inequality:

$$\begin{aligned} log\, J(\boldsymbol{\theta}') &= log \int_T \frac{p_{\boldsymbol{\theta}}(\boldsymbol{\tau})}{p_{\boldsymbol{\theta}}(\boldsymbol{\tau})} p_{\boldsymbol{\theta}'}(\boldsymbol{\tau})R(\boldsymbol{\tau})d\boldsymbol{\tau} \\ &\geq \int_T p_{\boldsymbol{\theta}}(\boldsymbol{\tau})R(\boldsymbol{\tau})log\, \frac{p_{\boldsymbol{\theta}'}(\boldsymbol{\tau})}{p_{\boldsymbol{\theta}}(\boldsymbol{\tau})}d\boldsymbol{\tau} \\ = -&\int_T p_{\boldsymbol{\theta}}(\boldsymbol{\tau})R(\boldsymbol{\tau})log\, \frac{p_{\boldsymbol{\theta}}(\boldsymbol{\tau})}{p_{\boldsymbol{\theta}'}(\boldsymbol{\tau})}d\boldsymbol{\tau} + const \end{aligned} \tag{6}$$

Therefore, it is easily observed that Equation 6 is proportional to $L(\boldsymbol{\theta}') = -D(p_{\boldsymbol{\theta}}(\boldsymbol{\tau})R(\boldsymbol{\tau})||p_{\boldsymbol{\theta}'}(\boldsymbol{\tau}))$. Then maximizing $L(\boldsymbol{\theta}')$ is mathematically equal to find the best update path for maximizing the reward expectation $J(\boldsymbol{\theta}')$. After differentiating $J(\boldsymbol{\theta}')$ with respect to $\boldsymbol{\theta}'$, we can obtain:

$$\begin{aligned} \partial_{\boldsymbol{\theta}'}L(\boldsymbol{\theta}') &= \int_T p_{\boldsymbol{\theta}}(\boldsymbol{\tau})R(\boldsymbol{\tau})\partial_{\boldsymbol{\theta}'}p_{\boldsymbol{\theta}'}(\boldsymbol{\tau})d\boldsymbol{\tau} \\ &= E\left\{(\sum_{t=1}^{T}\partial_{\boldsymbol{\theta}'}log\,\pi(\boldsymbol{a_t}|\boldsymbol{s_t},t))R(\boldsymbol{\tau})\right\} \end{aligned} \tag{7}$$

where $\pi(\boldsymbol{a_t}|\boldsymbol{s_t},t))$ is a policy following a norm Gaussian distribution. For the episodic case, we can simply say $R(\boldsymbol{\tau}) = Q(\boldsymbol{a_t}, \boldsymbol{s_t}) = \sum_{t=1}^{T}\lambda_t r_t$ where $Q(\boldsymbol{a_t}, \boldsymbol{s_t})$ is the action state function. $\lambda_t$ and $r_t$ are the discounting factor and the reward. In order to solve the $\boldsymbol{\theta}' = argmaxL(\boldsymbol{\theta}')$, Equation 7 is set to 0 when using the state-dependent actor. Therefore, assume the policy $\pi(\boldsymbol{a_t}|\boldsymbol{s_t},t)) = (2\pi\boldsymbol{\psi}^T\boldsymbol{\Sigma}\boldsymbol{\psi})^{-1/2}exp(\frac{(-a-\boldsymbol{\theta}^T\boldsymbol{\psi})^2}{2\boldsymbol{\psi}^T\boldsymbol{\Sigma}\boldsymbol{\psi}})$ and substitute this into Equation 7, we can get the solution to solve $\boldsymbol{\theta}'$:

$$E\left\{\sum_{t=1}^{T}\frac{((\boldsymbol{\theta}+\boldsymbol{\epsilon}_t)^T\boldsymbol{\psi})\boldsymbol{\psi}^T}{\boldsymbol{\psi}^T\Sigma\boldsymbol{\psi}}\right\} = E\left\{\sum_{t=1}^{T}\frac{\boldsymbol{\theta}'\boldsymbol{\psi}^T}{\boldsymbol{\psi}^T\Sigma\boldsymbol{\psi}}Q^\pi\right\} \tag{8}$$

Then Equation 8 yields:

$$\boldsymbol{\theta}' = \boldsymbol{\theta} + E\left\{\sum_{t=1}^{T}WQ^\pi\right\}^{-1} E\left\{\sum_{t=1}^{T}W\boldsymbol{\epsilon}_t Q^\pi\right\} \tag{9}$$

where $W = \boldsymbol{\psi}\boldsymbol{\psi}^T(\boldsymbol{\psi}^T\Sigma\boldsymbol{\psi})^{-1}$. When the $\Sigma$ is a diagonal as well as kept as a constant and $\boldsymbol{\psi}$ are normalized basis functions, Equation 9 can be simplified to:

$$\boldsymbol{\theta}'_i = \boldsymbol{\theta}_i + \frac{E\left\{\sum_{t=1}^{T}\sigma_i^2\epsilon_{i,t}Q^\pi\right\}}{\sum_{t=1}^{T}\sigma_i^2 Q^\pi} \tag{10}$$

where $\boldsymbol{\theta}'_i$ , $\boldsymbol{\theta}_i$, $\epsilon_{i,t}$ are the $i$th element of the correspondent vectors. In our work, we use Equation 10 since DMPs are used as the normalized basis functions.

## References


[1] M. Golubitsky and I. Stewart, *The Symmetry Perspective: From Equilibrium to Chaos in Phase Space and Physical Space*, ser. Progress in Mathematics. Birkh[Ux00e4]user, 2003.

[2] J. Peters, "Machine learning for motor skills in robotics," Ph.D. dissertation, University of Southern California, 2007.

[3] P. Dayan and G. E. Hinton, "Using expectation-maximization for reinforcement learning," *Neural Comput.*, vol. 9, no. 2, pp. 271–278, Feb. 1997.
